# Extended Kalman Filter-Based Visual Odometry in Dynamic Environments Using Modified 1-Point RANSAC

**DOI:** 10.3390/biomimetics10100710

**Published:** 2025-10-20

**Authors:** Jinhee Lee, Jaeyoung Kang

**Affiliations:** Department of Mechanical Engineering, Inha University, Incheon 22212, Republic of Korea; jinhee@inha.edu

**Keywords:** visual odometry, Extended Kalman Filter, computer vision

## Abstract

Visual odometry in dynamic environments is particularly challenging, as moving objects often cause incorrect data associations and large pose estimation errors. Traditional EKF-based VO methods rely on 1-point RANSAC to reject outliers under the assumption of a static world, thereby discarding dynamic landmarks as noise. However, in practice, outliers may arise not only from measurement errors but also from the motion of objects. To address this issue, we propose a modified 1-point RANSAC framework that detects dynamic objects and leverages both static and dynamic landmarks for ego-motion estimation. Inspired by adaptive strategies observed in biological vision systems, our approach integrates EKF-based state estimation with dynamic object tracking to achieve simultaneous ego-motion and object-motion estimation, improving robustness in complex and dynamic scenes.

## 1. Introduction

Visual odometry (VO) is a method that estimates the ego-pose, i.e., the position and orientation of an agent, using image data, and it has been widely applied in autonomous driving, robotics, and drone navigation. The fundamental concept underlying VO is to infer the agent’s relative motion by analyzing the changes in visual observations across consecutive frames. Similar mechanisms are observed in biological vision, where animals and insects estimate self-motion from optic flow and the relative motion of surrounding objects. Gay et al. [1] presented a navigation model inspired by place and grid cells, and Wang et al. [2] proposed REVIO using event-based vision inspired by retinal ganglion cells. Based on this concept, ego-pose estimation methods can be categorized into three types depending on the nature of data association: 2D-2D, 2D-3D (or equivalently, 3D-2D), and 3D-3D matching [3]. In 2D-2D matching, the relative pose between consecutive frames is estimated by analyzing geometric constraints between 2D feature correspondences extracted from consecutive images and is commonly used in monocular visual odometry [4], but it suffers from scale ambiguity, as depth information is not directly available. In 2D-3D matching, 2D image features are associated with known 3D landmarks obtained via triangulation (e.g., from stereo cameras) or direct depth sensors (e.g., RGB-D). The camera pose can then be estimated by solving the Perspective-n-Point (PnP) problem [5,6]. Finally, 3D-3D matching estimates the sensor’s motion by aligning two consecutive 3D point clouds. Since both point sets are defined in a common metric space, this approach inherently provides metric scale and high-precision motion estimation. It is widely employed in LiDAR-based and stereo camera-based ego-pose estimation pipelines, particularly where depth sensing is reliable and dense [7]. Similar mechanisms are observed in biological vision, where animals and insects estimate self-motion from optic flow and the relative motion of surrounding objects.

Beyond data association types, ego-pose estimation methods can also be classified by the underlying optimization framework used for state estimation. Among these, a common approach is to directly solve a bundle adjustment (BA) [8] problem over a sliding window of keyframes, refining both camera poses and landmarks by minimizing reprojection error. Alternatively, filtering-based methods such as the Extended Kalman Filter (EKF), and graph-based optimization techniques that model the problem as a factor graph, are also widely adopted.

EKF-based methods estimate the current state sequentially by propagating and updating the state vector and covariance using probabilistic models [9]. They are suitable for real-time applications due to their computational efficiency but may suffer from linearization errors and filter inconsistency in highly nonlinear environments. In contrast, graph-based optimization methods formulate the problem as a global nonlinear least-squares optimization over a factor graph, allowing for more accurate and globally consistent solutions by jointly optimizing all poses and measurements [10]. However, they are generally more computationally demanding than filtering methods, and may require additional engineering to meet strict real-time constraints. In recent years, graph-based methods have become dominant in VO and SLAM (Simultaneous Localization and Mapping) because they enable loop closure and globally consistent optimization [11,12,13]. Although EKF-based ego-pose estimation has been less actively studied in recent years, it still offers advantages such as low computational load, real-time recursive updates, and suitability for sensor fusion [14,15].

The presence of measurement noise and outliers significantly affects estimation accuracy. Several approaches have been proposed to distinguish outliers. In graph-based methods, robust graph-based optimization has been widely studied to mitigate the effects of outliers and measurement noise in visual and pose graph SLAM. Huber loss and other M-estimators have been commonly used to suppress the influence of large residuals in the cost function, providing robustness against occasional false matches in data association [16]. Dynamic Covariance Scaling (DCS) further improves robustness by down-weighting erroneous constraints based on their residual magnitudes in a closed-form, enabling efficient real-time operation without introducing additional variables [17]. Switchable Constraints (SC), on the other hand, explicitly model the reliability of each loop closure by introducing a latent switch variable that can deactivate incorrect constraints during optimization, achieving high resilience in the presence of false positives [18]. In EKF-based methods, Joint Compatibility Branch and Bound (JCBB) [19] is used to reject outliers in ego-pose estimation by evaluating all possible measurement associations and selecting the largest subset that meets joint statistical consistency criteria. Compared to JCBB, which performs a full branch-and-bound search over all possible data association hypotheses and selects the largest jointly compatible inlier set, 1-point RANSAC [20] significantly reduces computational cost by generating motion hypotheses from a single randomly selected feature (or landmark), estimating ego-pose through EKF, and identifying inlier features by thresholding their innovation residuals.

Since general algorithms are assumed to be used in static environments, their performance degrades when dynamic objects are present, leading to incorrect data associations and large pose estimation errors. To address this issue, robust outlier rejection should be applied for ego-pose estimation in dynamic environments. Cao et al. [21] propose a method that integrates optical flow-based motion state estimation into the sliding window optimization of VINS-Mono, enabling robust visual-inertial odometry in dynamic environments without relying on semantic information. Likewise, Li et al. [22] enhance sliding-window BA VIO by incorporating IMU-PARSAC-based matching and robust pure-rotation handling. Ballester et al. [23] integrate segmentation and photometric optimization into ORB-SLAM2 to remove dynamic features and perform BA-based optimization. Similarly, Wu et al. [24] extend ORB-SLAM3 with optical flow segmentation to eliminate dynamic outliers before BA optimization. Ram [25] adds planar homography constraints to the BA framework of VINS-Mono to achieve stable optimization under dynamic conditions. As an EKF-based method, Eckenhoff et al. [26] propose a Schmidt-EKF that uses dynamic object features only for tracking the object itself, while preventing them from directly updating the ego-motion estimate, thus maintaining robustness under inaccurate target motion models. Also, Ma et al. [27] propose an IEKF framework that applies Schur complement-based marginalization to efficiently eliminate visual features from the state vector, improving scalability and accuracy. On the other hand, as a graph-based method, Song et al. [28] propose a dynamic-aware VIO system that suppresses the influence of dynamic features by adaptively reweighting their motion residuals based on Black-Rangarajan duality, without explicitly classifying or rejecting them. Song et al. [29] extend this approach by introducing Adaptive Truncated Least Squares to fully eliminate the effect of outliers and incorporate bias correction mechanisms for handling abruptly dynamic objects. Liu et al. [30] proposed IPL-SLAM, which filters dynamic features using instance segmentation and motion consistency checks while framing the fusion of semantic and geometric cues as a biologically inspired perception strategy. In these works, dynamic features are effectively excluded from ego-pose estimation through robust optimization. In natural vision, dynamic elements are not always regarded as noise but often provide informative cues for navigation. This perspective motivates the incorporation of dynamic landmarks into VO rather than discarding them.

The aforementioned methods reject outlier features detected from dynamic objects so that the effect of measured features gets lower and they will be not suitable in highly dynamic environments. Several studies have explored ways to incorporate dynamic features into ego-pose estimation rather than simply excluding them. As an EKF-based approach, Todoran et al. [31] propose an EKF-based SLAM framework that models dynamic objects as individual state variables, allowing the system to incorporate their motion into ego-pose estimation rather than discarding them as outliers. This object-level approach improves robustness in dynamic environments by leveraging stable object motion for pose correction. However, the method is sensitive to segmentation quality and association accuracy because pose updates are performed only after segments are grouped and tracked as coherent objects. As another approach of EKF-based method, Chiu [32] proposes a unified optimization-based framework that jointly estimates ego-pose, static landmarks, and dynamic object trajectories using both static and dynamic features. This approach integrates localization, mapping, and multi-object tracking within a single EKF-based backend, offering a coherent treatment of dynamic scenes. However, the method does not incorporate explicit outlier rejection, making it vulnerable to erroneous dynamic feature measurements. The evaluation is limited to simulation, and no mechanism is provided to ensure robustness under real-world perception errors. As a graph-based approach, Tian et al. [33] propose DL-SLOT, a dynamic LIDAR SLAM and object tracking framework that jointly estimates the ego-pose and the states of stationary and dynamic objects within a unified sliding window-based collaborative graph optimization. Also, Huang et al. [34] present a graph-based backend that explicitly clusters landmarks into rigid bodies, jointly estimates both ego-pose and per-object motions through decoupled factor graph optimization, and thereby mitigates the negative impact of dynamic objects on pose estimation without discarding their measurements as outliers.

Previous EKF-based methods that use dynamic landmarks for estimation did not provide a clear strategy for outlier rejection in dynamic environments, making the implementation of VO difficult in practice [31,32]. Conventional 1-point RANSAC assumes that all features belong to a static scene and thus rejects those affected by independently moving objects, resulting in the loss of informative visual cues and degraded pose estimation in dynamic environments. To address this limitation, we propose a novel EKF-based VO methodology that modifies 1-point RANSAC to detect dynamic objects and incorporate both static and dynamic landmarks into ego-pose estimation. A supplementary video shows the evaluation of the proposed method, including comparisons with classical methods (Video S1). This design is consistent with a bio-inspired perspective, where dynamic visual cues are treated as informative signals rather than discarded as outliers, reflecting adaptive strategies observed in biological perception. This paper focuses on applications for vehicle navigation with a stereo camera, and the main contributions are as follows:

(1) A new algorithm of EKF-based visual odometry using stereo camera is proposed, which utilizes both static and dynamic landmarks for ego-pose estimation. This method uses instance segmentation to detect and track objects from image, so that it can use the characteristics of image. Also, in multiple object tracking on road, our method is robust to truncation due to angle of view, which often causes a shift in the observed point cloud centroid as the object moves near the boundary of the field of view.
(2) Reasonable outlier rejection in dynamic environment is conducted by 1-point RANSAC and RANSAC-based transformation estimation from point cloud. The method of outlier rejection and determining whether an object is static or dynamic is proposed.
(3) Tests are conducted using real-world image dataset to evaluate the performance of ego-pose estimation in dynamic environment with noise.

This paper is organized as follows. In Section 2, overall structure of the modified 1-point RANSAC for dynamic environments is proposed. In Section 3, evaluations are conducted to determine whether the proposed algorithm is suitable for estimating ego-pose in dynamic environments. Finally, in Section 4, conclusions are stated.

## 2. Modified 1-Point RANSAC for Dynamic Environments

In this section, the overall structure and the detailed methods of the proposed algorithm using both static and dynamic landmarks assuming dynamic environments is introduced. Since EKF is employed for the estimation, the state transition model and the measurement model used in the filter are first introduced. The proposed algorithm is divided into 3 steps: (1) measurement by stereo camera, (2) the classical 1-point RANSAC algorithm assuming static environment, (3) additional steps for considering dynamic landmarks. Since the proposed method incorporates the classical method, before introducing the ego-pose estimation method assuming dynamic environments, classical 1-point RANSAC algorithm which is assuming static environments is briefly introduced to define variables and facilitate comparison with the modified approach. Then, detailed methods for state estimation of dynamic objects and augmented estimation of ego-pose and landmark positions used in the proposed algorithm are further explained.

### 2.1. Models for EKF

In this paper, in the EKF-based VO framework, the ego-pose evolves according to the state transition model,

(1) $$\xi_{k}=g\left(\xi_{k-1},u_{k}\right)+q_{\xi },$$

where $\xi_{k}≜{\left[x,y,\psi \right]}_{k}^{⊤}$ denotes the ego-pose of the vehicle in the global coordinates at current frame *k*, $u_{k}$ denotes the control input and $q_{\xi }∼N\left(0,Q_{\xi }\right)$ denotes the state noise of the ego-pose. $x≜{\left[x,y\right]}^{⊤}$ denotes the ego position on the road plane, and ψ denotes the ego heading on road plane. In this paper, the bicycle model is employed as the state transition model g.

The positions of landmarks are assumed to be static in the classic 1-point RANSAC, the state transition of the landmark position is represented as

(2) $$X_{k}^{i}=X_{k-1}^{m(i)}+q_{X},$$

where $X_{k}^{i}≜{\left[X,Y\right]}_{k}^{i⊤}$ denotes the position on the 2D road plane of *i*-th landmark at the *k*-th frame in the global coordinates, m(i) denotes the index of the associated landmark in the previous frame, and $q_{X}∼N\left(0,Q_{X}\right)$ denotes the state noise of landmark position which is assumed to have Gaussian distribution.

The landmark measurements are represented in 2D polar coordinates relative to the camera coordinate system, so that the relationship between measurements and states is denoted as

(3) $$\begin{array}{cc} \; & z_{k}^{i}≜{\left[\rho ,\theta \right]}_{k}^{i⊤}=h\left(X_{k}^{i},\xi_{k}\right)+r_{z,k}^{i},\\ \; & {\hat{z}}_{k}^{i}=h\left(X_{k}^{i},\xi_{k}\right)=\left[\begin{array}{c} \sqrt{{\left(X_{k}^{i}-x_{k}\right)}^{2}+{\left(Y_{k}^{i}-y_{k}\right)}^{2}}\\ \arctan2\left(Y_{k}^{i}-y_{k},X_{k}^{i}-x_{k}\right)-\psi_{k} \end{array}\right], \end{array}$$

where $z_{k}^{i}$ denotes the measurement of the *i*-th landmark position relative to the camera coordinate system, ρ is the distance from the agent, θ is the bearing angle relative to the ego heading ψ, and $r_{z,k}^{i}∼N\left(0,R_{z}\left(z_{k}^{i}\right)\right)$ represents the measurement noise of landmark *i* at the *k*-th frame.

The polar representation is adopted to accurately model the characteristics of the measurements obtained in the vehicle body frame and to incorporate the uncertainty originating from stereo-based feature reconstruction. Because the landmark positions are defined relative to the vehicle, their measurements are directly affected by the vehicle’s rotational noise, and such relationships are more conveniently expressed in polar coordinates than in Cartesian form. The range-dependent uncertainty of stereo-reconstructed feature points caused by disparity and pixel localization errors can be naturally incorporated into the EKF measurement covariance by expressing the measurements in polar coordinates, thereby improving the consistency of state estimation.

In this paper, the covariance of landmark measurement is not assumed to follow a Gaussian distribution with constant variance, but is instead assumed as a function of the radial distance ρ and angle θ of the landmark measurement, represented as

(4) $$R_{z}\left(z_{k}^{i}\right)≜diag\left(\sigma_{\rho }^{2}\left(\rho_{k}^{i}\right),\sigma_{\theta }^{2}\left(\theta_{k}^{i}\right)\right),$$

(5) $$\begin{array}{c} \sigma_{\rho }^{2}\left(\rho_{k}^{i}\right)≜\alpha_{\rho }{\left(\rho_{k}^{i}\right)}^{4}\exp\left(-\beta_{\rho }S_{\Delta f,k}^{i}\right)\\ \sigma_{\theta }^{2}\left(\theta_{k}^{i}\right)≜\alpha_{\theta }\cos^{4}\left(\theta_{k}^{i}\right)\exp\left(-\beta_{\theta }S_{f,k}^{i}\right), \end{array}$$

where α and β denotes the constant parameters of each measurement, $S_{\Delta f}$ denotes the score of feature matching and $S_{f}$ denotes the score of feature detection. The variance is designed to decrease with increasing score through the use of an exponential function. In our assumption, the variance of radial distance ρ is proportional to $\rho^{4}$ and the variance of angle θ is proportional to $\cos^{4}\left(\theta \right)$. A detailed derivation supporting this assumption is discussed in Appendix D. The augmented state evolves according to the state transition model,

(6) $$\chi_{k}=f\left(\chi_{k-1},u_{k}\right)+q,\chi_{k}≜\left[\begin{array}{c} \xi_{k}\\ X_{k}^{i},\forall i\in I_{k} \end{array}\right],$$

where $\chi_{k}$ denotes the total state that contains ego-pose and landmark positions, q denotes the noise of total state, and $I_{k}$ denotes the set of inlier indices at *k*-th frame. The augmented state transition model is used for the final EKF update, denoted as

(7) $$f\left(\chi_{k-1},u_{k}\right)=\left[\begin{array}{c} g(\xi_{k-1},u_{k})\\ X_{k-1}^{m(i)},\forall i\in I_{k} \end{array}\right].$$

### 2.2. Classical 1-Point RANSAC Assuming Static Environments

**Figure 1:**
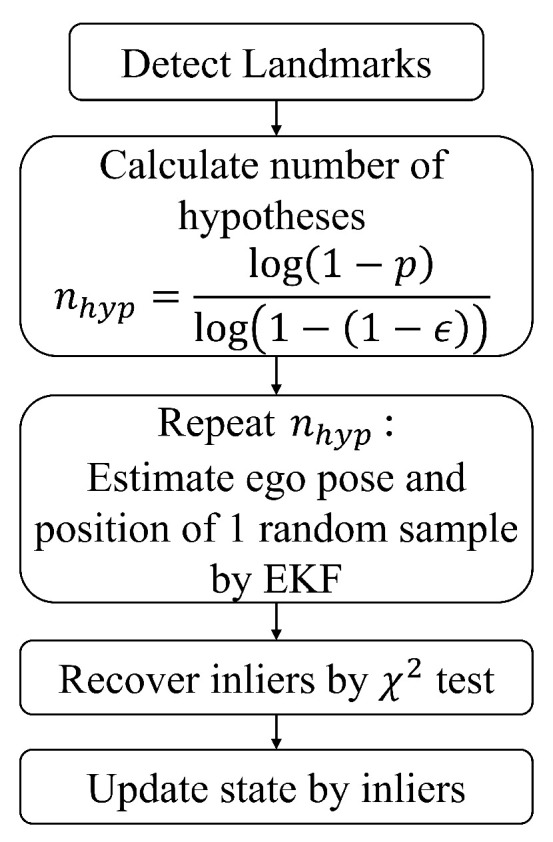
Pipeline of 1-point RANSAC algorithm.

Figure 1 indicates the pipeline of the classical 1-point RANSAC algorithm and detailed procedure is represented in Appendix A. A random landmark $z_{k}^{h}$ is selected as sample for the hypothesis and a temporary state update is executed using only one sample. This procedure is repeated $n_{\mathrm{hyp}}$ times to find a sample which has the most consensus to maximize the number of inliers. $n_{\mathrm{hyp}}$, the number of hypothesis repetition, is determined to guarantee that at least one hypothesis is generated from an inlier with a probability *p*, given an estimated inlier ratio ϵ. The equation of $n_{\mathrm{hyp}}$ is represented as

(8) $$n_{\mathrm{hyp}}=\frac{\log(1-p)}{\log\left(1-{\left(1-\epsilon \right)}^{1}\right)}.$$

During each RANSAC iteration, landmarks with innovation below the threshold are considered inliers. Specifically, after performing the temporary state update using a randomly selected landmark, the predicted measurements of all other landmarks are computed, and their innovations $\nu_{j}=z_{k}^{j}-{\hat{z}}_{k}^{j}$ are evaluated. Each feature $j\in J_{k}$, where $J_{k}$ denotes the set of valid landmarks observed at time step *k* (see Section 2.3.1), is classified as an inlier if its innovation magnitude satisfies $∥\nu_{j}∥<\gamma_{\mathrm{innov}}$, where $\gamma_{\mathrm{innov}}$ is a predefined innovation threshold. Based on each hypothesis, an inlier set $I^{h}$ is constructed. If the number of inliers, $|I^{h}|$, exceeds that of the best inlier set found so far, the best inlier set $\tilde{I}$ and the corresponding hypothesis for the ego-pose and landmarks are updated accordingly. This process is repeated until the number of evaluated hypotheses reaches the predefined maximum $n_{\mathrm{hyp}}$.

The selected inlier set $\tilde{I}$ is then used to perform a full EKF update as part of the high-innovation inliers recovery procedure, resulting in an updated state estimate ${\hat{\chi }}_{k|k}$ that includes both the ego-pose and the inlier landmark positions.

After this update, all remaining candidate features $i\in J_{k}\setminus \tilde{I}$ are re-evaluated against the updated state using the Mahalanobis distance,

$$d_{i}=\nu_{i}^{⊤}S_{i}^{-1}\nu_{i},$$

where $\nu_{i}$ is the innovation and $S_{i}$ the corresponding innovation covariance.

A feature *i* is additionally classified as an inlier if $d_{i}<\gamma_{\chi^{2}}$, where $\gamma_{\chi^{2}}$ is the chi-square threshold. Accordingly, the final inlier set is defined as

(9) $$I_{k}=\tilde{I}\cup \left\{i\in J_{k}\setminus \tilde{I}\;|\;d_{i}<\gamma_{\chi^{2}}\right\}.$$

### 2.3. Modified 1-Point RANSAC Assuming Dynamic Environments

**Figure 2:**
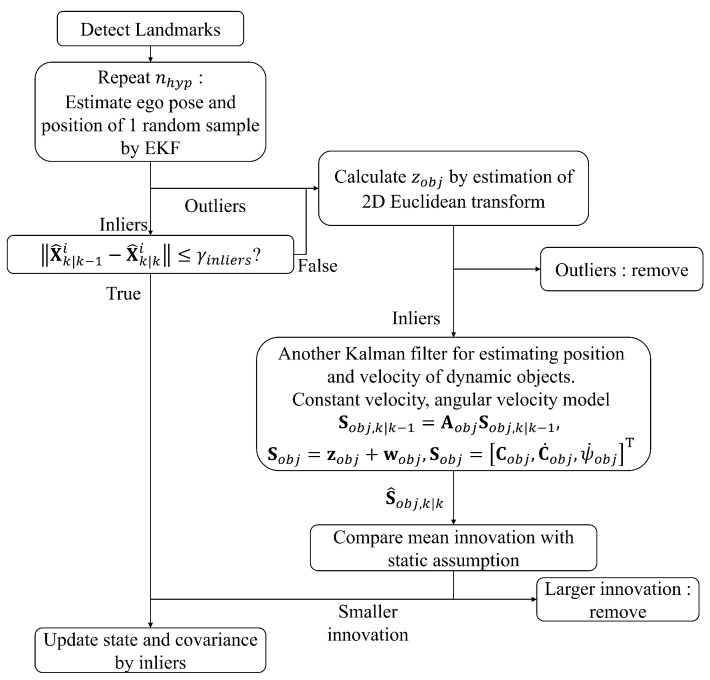
Overall structure of the modified 1-point RANSAC algorithm.

In the proposed EKF-based framework, landmarks belonging to dynamic objects are not rejected but incorporated into the estimation process, considering the presence of dynamic environments. Figure 2 illustrates the overall structure of the proposed algorithm.

The proposed algorithm consists of five main stages, as summarized below. Each stage corresponds to the detailed procedures presented in Section 2.3.1, Section 2.3.2, Section 2.3.3, Section 2.3.4 and Section 2.3.5.

(1) **Measurement (Section 2.3.1):** Landmark positions are reconstructed in the camera coordinate system from stereo image pairs. Instance segmentation is then applied to classify features into object and background regions.
(2) **Classical 1-point RANSAC (Section 2.3.2):** Matched landmarks between consecutive frames are processed using the standard 1-point RANSAC to classify inliers and outliers. Outliers, as well as inliers with large innovation, are treated as potentially dynamic features. The estimated ego-pose at this stage serves as a temporary estimate for the following steps.
(3) **Object motion estimation (Section 2.3.3):** The rigid-body motion of each detected object is estimated from its point cloud using a RANSAC-based Euclidean transform. A secondary Kalman filter is then applied to refine the state of each object using the estimated transformation.
(4) **Augmented state estimation (Section 2.3.4):** The ego-pose and landmark states are jointly updated using inliers from both static and dynamic landmarks within the EKF framework, enabling consistent estimation under dynamic scenes.
(5) **Mapping of newly detected landmarks (Section 2.3.5):** Newly observed or unmatched landmarks are initialized and incorporated into the map using the filtered ego-pose.

#### 2.3.1. Feature Detection, Matching and Multiple Object Tracking

**Figure 3:**
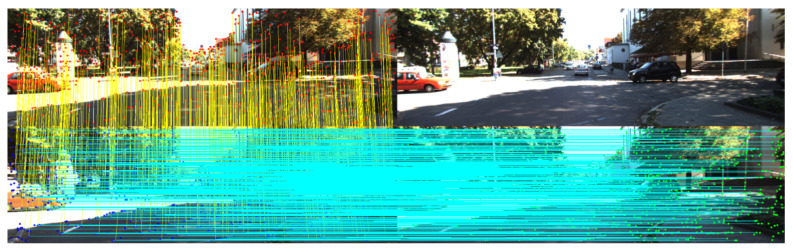
Feature detection and matching visualization. (**Top**): previous stereo images (left and right) with detected features and object masks. (**Bottom**): current stereo images. Feature correspondences are shown between the previous and current left images (temporal matching, left column), and between the current left and right images (stereo matching, bottom row).

As visual measurements, features in pixel coordinates are detected and matched using a stereo camera. In this paper, SuperPoint [35] is employed for feature detection, and SuperGlue [36] is used for feature matching. Deep learning-based feature detection and matching are adopted to enhance feature matching reliability in outdoor environments where repetitive patterns such as windows, tiles, or crosswalks often cause correspondence errors, as the graph neural network in SuperGlue effectively leverages spatial relationships between keypoints to maintain robust matches under such conditions. The feature detector extracts keypoints from the left stereo image, and the feature matching algorithm finds correspondences with features in the right image based on those keypoints. The matched feature coordinates in the left and right images are represented as

(10) $$\begin{array}{cc} \; & F_{k}≜{\left\{f_{k}^{i}≜{\left[v,w\right]}_{k}^{i⊤}\right\}}_{i=1}^{N_{f}},\\ \; & F_{k}^{*}≜{\left\{f_{k}^{*i}≜{\left[v^{*},w^{*}\right]}_{k}^{i⊤}\right\}}_{i=1}^{N_{f}}, \end{array}$$

where $F_{k}$ and $F_{k}^{*}$ denote the sets of 2D pixel coordinates of $N_{f}$ matched features in the left and right stereo images, respectively. The left features with valid correspondences in the right image are also matched to the left image of previous frame, k−1. Two set of already calculated pixel points are matched by feature matching algorithm, in contrast to the matching with the right image, that the features are tracked to define the pixel position in the right image. The left features with valid correspondences in the right image and the previous frame are used to estimate ego-pose and the set of valid features is denoted as $J_{k}$, and a set of new features with valid correspondences in the right image but unvalid correspondences in the previous frame is denoted as $J_{k}^{*}$ Figure 3 indicates the procedure of the feature measurement. (Below: stereo image at current frame, Upper: stereo image at previous frame)

The 3D positions of landmarks in the camera coordinates, ${\overset{´}{X}}_{k}^{i}$, are obtained via stereo triangulation for each inlier feature $i\in I_{k}$. These positions are then converted to 2D polar coordinates relative to the camera, denoted as $z_{k}^{i}$, which serve as the measurement inputs to the filter. Landmarks are classified according to the objects by calculating which segmentation mask each feature point is located in. The set of landmark indices located in object *o* is denoted as $P_{k}^{o}$. Also, the objects are assigned IDs and tracked using a MOT (Multiple Object Tracking) algorithm, so that each object’s ID can be matched with its corresponding instance from previous frames. YOLOv8 is used in the proposed algorithm to perform instance segmentation, and is integrated with the MOT algorithm ByteTrack [37] for object tracking.

#### 2.3.2. 1-Point RANSAC Assuming Static Environment

The classical 1-point RANSAC, assuming static environment, is conducted using valid landmark positions $\left\{z_{k}^{i}|i\in J_{k}\right\}$, while the high-innovation inliers recovery procedure is excluded. The best inlier set $\tilde{I}$ is considered directly as the final inlier set $I_{k}$. The temporarily filtered ego-pose ${\hat{\xi }}_{k|k}^{-}$ is calculated, and landmarks are divided into inliers and outliers. Unlike the outliers are just rejected in classic method, in the proposed method, they are considered potentially dynamic landmarks which have the opportunity to be used in the estimation.

When an object is considered to be potentially dynamic, it is assumed to have either outlier landmarks or landmarks that pass the innovation threshold but still exhibit motion. Since some dynamic landmarks can be mistakenly included as inliers by 1-point RANSAC due to their small innovation, the distance between each landmark’s current filtered position and its previous position is computed to detect subtle motion. Whether a landmark is considered potentially dynamic depends on whether the object it belongs to is determined to be dynamic.

To identify such cases, a subset of landmarks that passed the innovation threshold $\gamma_{\mathrm{innov}}$ but exhibit a significant positional change is extracted for each object. This set is defined as

(11) $$D_{1,k}^{o}=\left\{i\in I_{k}\cap P_{k}^{o}\;|\;∥{\hat{X}}_{k|k}^{i}-{\hat{X}}_{k|k-1}^{i}∥>\gamma_{\mathrm{inliers}}\right\},$$

where $I_{k}$ denotes the inlier set and $P_{k}^{o}$ denotes the set of landmarks belonging to object *o*.

If the proportion of such landmarks for object *o* exceeds a predefined threshold, the object is classified as dynamic. The set of such objects is denoted as

(12) $$O_{1,k}=\left\{o\;|\;\frac{|D_{1,k}^{o}|}{|P_{k}^{o}|}>\gamma_{\mathrm{ratio}}\right\}.$$

Next, landmarks that were rejected as outliers by the 1-point RANSAC procedure and failed the innovation threshold test are grouped per object, denoted as

(13) $$D_{2,k}^{o}=\left(J_{k}\setminus I_{k}\right)\cap P_{k}^{o}.$$

An object is also considered potentially dynamic if the ratio of outlier landmarks exceeds the threshold:

(14) $$O_{2,k}=\left\{o\;|\;\frac{|D_{2,k}^{o}|}{|P_{k}^{o}|}>\gamma_{\mathrm{ratio}}\right\}.$$

The total set of potentially dynamic objects is defined as the union:

$${\tilde{O}}_{k}=O_{1,k}\cup O_{2,k}.$$

For each object $o\in {\tilde{O}}_{k}$, the complete set of associated dynamic landmarks is given by:

$${\tilde{D}}_{k}^{o}=D_{1,k}^{o}\cup D_{2,k}^{o}.$$

#### 2.3.3. Estimation of Planar Motion of Dynamic Objects with Kalman Filter

Another Kalman filter is applied to each object *o* in the potentially dynamic object set ${\tilde{O}}_{k}$, in order to estimate the motions of dynamic objects. The Euclidean transformation of each object, consisting of the rotation $\Omega_{k}^{o}\in \mathrm{SO}\left(2\right)$ and the translation $\tau_{k}^{o}\in R^{2}$, is estimated by comparing the current point cloud with the previous one. The term $\Omega_{k}^{o}$ denotes the rotation, and $\tau_{k}^{o}$ denotes the translation of object *o* at frame *k*.

The estimation of transformation by comparison is conducted using raw point cloud for both current frame and previous frame, because the point cloud containing filtered landmarks is hard to estimate exact transformation. The position of each raw point at current frame in global coordinates is computed using the temporarily filtered ego-pose, denoted as

(15) $${\tilde{X}}_{k}^{i}=\Phi \left({\hat{\psi }}_{k|k}^{-}\right){\overset{´}{X}}_{k}^{i}+{\hat{x}}_{k|k}^{-}.$$

Similarly, the position of each raw point at previous frame in global coordinates is computed using the finally filtered ego-pose at previous frame, denoted as

(16) $${\tilde{X}}_{k-1}^{i}=\Phi \left({\hat{\psi }}_{k-1|k-1}\right){\overset{´}{X}}_{k-1}^{m(i)}+{\hat{x}}_{k-1|k-1},$$

where Φ(ψ)∈SO(2) denotes the rotation matrix of angle ψ.

When estimating the transformation of each point cloud, RANSAC is conducted to distinguish the inlier landmarks that fit with the estimated transform and the outliers that do not. The algorithm of RANSAC-based Euclidean transform estimation is indicated in Appendix B. As outlined in Algorithm A2, the method iteratively selects minimal pairs of point correspondences and estimates a candidate rigid trasformation using a least-squares solution as represented in Algorithm A3. For each hypothesis, the transformation is applied to all source points, and the reprojection error is computed. Inliers are determined by thresholding the error, and the transformation yielding the largest inlier set is selected as the final result. This procedure provides robustness against outliers and ensures that the estimated transformation best alignes the two point sets under Euclidean constraints.

Due to the possibility that instance segmentation may erroneously assign background pixels to object instances, the resulting point clouds can contain spurious points. To address this, additional filtering using point cloud clustering is performed to suppress such noise. DBSCAN (Density-Based Spatial Clustering of Applications with Noise) [38] is conducted to cluster the inliers of potentially dynamic point clouds. After performing point cloud clustering, the cluster containing the largest number of points is considered the representative cluster, while the remaining clusters are treated as noise and excluded from further computations. The set of representative landmark indices for each object *o* are denoted as $D_{k}^{o}$.

The measurement used in Kalman filter for object state, $\zeta_{k}^{o}$, is calculated using the estimated transformation, represented in Appendix C. While the center of the point cloud is primarily assumed to correspond to the object center, velocity and angular velocity are additionally considered to compensate for potential center shifts induced by occlusion.

The measurement of object state is denoted as

(17) $$\zeta_{k}^{o}=\left[\begin{array}{c} \frac{1}{\left|{\tilde{D}}_{k}^{o}\right|}\sum_{i\in {\tilde{D}}_{k}^{o}}{\tilde{X}}_{k}^{i}\\ \tau_{o}/\Delta t\\ \arctan2(\Omega {\left(2,1\right)}_{o},\Omega {\left(1,1\right)}_{o})/\Delta t \end{array}\right].$$

To ensure the reliability of object motion estimation, several validation steps are incorporated before accepting the estimated state as a valid measurement for the Kalman filter update. These checks are essential to eliminate ill-posed or noisy results that may compromise the accuracy of the estimation process. First, each object must be associated with at least three 3D points to enable rigid transformation estimation. Then, the rigid transformation matrix M between the previous and current point sets is estimated using a RANSAC-based method. The transformation is accepted only if it yields a non-empty set of inliers that support the estimated motion. From the estimated rigid transform M, the translation vector τ and rotation matrix Ω are extracted. Third, the spatial distribution of the inlier points is evaluated using their covariance matrix. If the maximum eigenvalue of this matrix exceeds a threshold $T_{\mathrm{cov}}$, indicating high spatial dispersion or unreliability, the object is discarded from further processing. An object is classified as an outlier and excluded from the Kalman filter update if it fails to satisfy any of the above criteria.

And the measurement model is represented as

(18) $$\zeta_{k}^{o}=s_{k}^{o}+r_{\zeta ,k}^{o}$$

where $s_{k}^{o}≜{\left[c,\dot{c},\dot{\phi }\right]}_{k}^{o⊤}$ denotes the state, $c_{k}^{o}≜{\left[\bar{X},\bar{Y}\right]}_{k}^{o⊤}$ denotes the position, ${\dot{\phi }}_{k}^{o}$ denotes the angular velocity of the point cloud of object *o* at *k*-th frame, and $r_{\zeta ,k}^{o}∼N\left(0,R_{\zeta }\left(\zeta_{k}^{o}\right)\right)$ denotes the measurement noise of object state.

The measurement error covariance $R_{\zeta }\left(\zeta_{k}^{o}\right)$ is calculated considering the number of point used to compute each value of the state, represented as

(19) $$R_{\zeta }\left(\zeta_{k}^{o}\right)=\frac{\sigma^{2}}{|{\tilde{D}}_{k}|}\cdot \mathrm{diag}\left(\mu_{\mathrm{pos}},\mu_{\mathrm{pos}},\mu_{\mathrm{vel}},\mu_{\mathrm{vel}},\mu_{\mathrm{angle}}\right)$$

Each object state is filtered assuming planar motion with constant velocity, constant anglular velocity, represented as

(20) $$s_{k}^{o}=As_{k-1}^{o}+q_{s},A=\left[\begin{array}{ccccc} 1 & 0 & \Delta t & 0 & 0\\ 0 & 1 & 0 & \Delta t & 0\\ 0 & 0 & 1 & 0 & 0\\ 0 & 0 & 0 & 1 & 0\\ 0 & 0 & 0 & 0 & 1 \end{array}\right]$$

where $q_{s}∼N\left(0,\Lambda \right)$ denotes the state error of objects.

#### 2.3.4. Augmented Estimation with Both Static and Dynamic Landmarks

Since the motions of the dynamic objects are estimated, both static and dynamic landmarks are ready to be used in estimation. Unlike the transition model of the static landmarks is same as mentioned in Equation (2), the transition model of each dynamic landmark is denoted as

(21) $$X_{k}^{i}=l\left(X_{k-1}^{m(i)},c_{k-1}^{o},{\dot{c}}_{k}^{o},{\dot{\phi }}_{k}^{o}\right)+\eta_{k}^{i},$$

(22) $$\begin{array}{c} l(X_{k-1}^{m(i)},c_{k-1}^{o},{\dot{c}}_{k}^{o},{\dot{\phi }}_{k}^{o})\\ ≜\Phi \left({\dot{\phi }}_{k}^{o}\Delta t\right)\left(X_{k-1}^{m(i)}-c_{k-1}^{o}\right)+c_{k-1}^{o}+{\dot{c}}_{k}^{o}\Delta t, \end{array}$$

where $\Phi \left({\dot{\phi }}_{k}^{o}\Delta t\right)\in \mathrm{SO}\left(2\right)$ denotes the rotation matrix of angle ${\dot{\phi }}_{k}^{o}\Delta t$ and $\eta^{i}$ denotes the state noise of landmark *i*.

When using dynamic landmarks to estimate ego-pose, it can cause more error than assuming static environment if there exists large error in object state estimation. To avoid the adverse effect of using the proposed algorithm, the vailidity of the estimation of the dynamic object states should be judged. Mean innovation of the estimated landmark positions of each object is calculated to judge the validity of each object denoted as

(23) $${\bar{\nu }}_{1,k}^{o}=\frac{1}{|{\tilde{D}}_{k}^{o}|}\sum_{i\in O_{k}^{o}}\left({\tilde{X}}_{k}^{i}-l\left({\hat{X}}_{k-1|k-1}^{m(i)},c_{k-1}^{o},{\dot{c}}_{k}^{o},{\dot{\phi }}_{k}^{o}\right)\right)$$

(24) $${\bar{\nu }}_{2,k}^{o}=\frac{1}{|{\tilde{D}}_{k}^{o}|}\sum_{i\in {\tilde{O}}_{k}^{o}}\left({\tilde{X}}_{k}^{i}-{\hat{X}}_{k-1|k-1}^{m(i)}\right)$$

(25) $$O_{k}=\left\{o\in {\tilde{O}}_{k}|{\bar{\nu }}_{1,k}^{o}<{\bar{\nu }}_{2,k}^{o}\right\}$$

The total transition model is represented as

(26) $$\begin{array}{c} f(\chi_{k-1},u_{k})=\\ \left[\begin{array}{c} g(\xi_{k-1},u_{k})\\ \left.\begin{array}{c} \forall i\in I_{k}\cup \underset{o\in O_{k}}{⋃}D_{k}^{o},\\ \;\left\{\begin{array}{cc} X_{k-1}^{m(i)} & \mathrm{if}\;i\in I_{k}\setminus \underset{o\in O_{k}}{⋃}D_{k}^{o}\\ l(X_{k-1}^{m(i)},\;c_{k-1}^{o},\;{\dot{c}}_{k}^{o},\;{\dot{\phi }}_{k}^{o}) & \mathrm{if}\;i\in D_{k}^{o},\;o\in O_{k} \end{array}\right. \end{array}\right. \end{array}\right] \end{array}$$

The state transition model $f(\chi_{k-1},u_{k})$ jointly updates the ego-pose and the landmark positions as components of an augmented state vector. The ego-pose is propagated using the motion model $g(\xi_{k-1},u_{k})$, while the landmarks are updated based on whether they are associated with dynamic objects or not. Dynamic landmarks are propagated according to the estimated motion of their corresponding object, whereas static ones retain their previous positions.

The Jacobian of the total transition model is also represented as

(27) $$\begin{array}{c} F(\chi_{k-1},u_{k})=\\ \mathrm{blockdiag}\left(G\left(\xi_{k-1},u_{k}\right),\begin{array}{c} \forall i\in I_{k}\cup \underset{o\in O_{k}}{⋃}D_{k}^{o},\\ \left\{\begin{array}{cc} I & \mathrm{if}\;i\in I_{k}\setminus \underset{o\in O_{k}}{⋃}D_{k}^{o}\\ \Phi ({\dot{\phi }}_{k}^{o}\Delta t) & \mathrm{if}\;i\in D_{k}^{o},\;o\in O_{k} \end{array}\right. \end{array}\right) \end{array}$$

The Jacobian of the state transition model, $F(\chi_{k-1},u_{k})$, is represented in block-diagonal form. It consists of the ego-pose Jacobian $G(\xi_{k-1},u_{k})$ and landmark-specific blocks, where each block is set to the identity matrix for static landmarks, and to a rotation matrix $\Phi ({\dot{\phi }}_{k}^{o}\Delta t)$ for dynamic landmarks, depending on whether $i\in D_{k}^{o}$ for some $o\in O_{k}$.

The update of total state is updated following the standard EKF procedures, represented as

(1) State Prediction ${\hat{\chi }}_{k|k-1}=f\left({\hat{\chi }}_{k-1|k-1},u_{k}\right)$
(2) Covariance Prediction $P_{k|k-1}=F_{k}P_{k-1|k-1}F_{k}^{⊤}+Q_{k}$
(3) Residual $\nu_{k}=z_{k}-h\left({\hat{x}}_{k|k-1}\right)$
(4) Innovation Covariance $S_{k}=H_{k}P_{k|k-1}H_{k}^{⊤}+R_{k}$
(5) Kalman Gain $K_{k}=P_{k|k-1}H_{k}^{⊤}S_{k}^{-1}$
(6) State Update ${\hat{\chi }}_{k|k}={\hat{\chi }}_{k|k-1}+K_{k}\nu_{k}$
(7) Covariance Update $P_{k|k}=\left(I-K_{k}H_{k}\right)P_{k|k-1}$

#### 2.3.5. Mapping of Newly Detected Landmarks

Mapping of newly detected landmarks, $\left\{{\overset{´}{X}}_{k}^{i}|i\in J_{k}^{*}\right\}$, is conducted in final sequence. The positions of the newly detected landmarks is calculated using ego-pose state, denoted as

(28) $${\tilde{X}}_{k}^{i}=\Phi \left({\hat{\psi }}_{k|k}\right){\overset{´}{X}}_{k}^{i}+{\hat{x}}_{k|k}$$

Mapping of newly detected landmarks, $\left\{{\overset{´}{X}}_{k}^{i}|i\in J_{k}^{*}\right\}$, is conducted at the final time step. Each landmark position, originally represented in the camera coordinate system, is transformed into the global coordinate frame using the estimated ego-pose:

(29) $${\tilde{X}}_{k}^{i}=\Phi \left({\hat{\psi }}_{k|k}\right){\overset{´}{X}}_{k}^{i}+{\hat{x}}_{k|k},$$

where ${\tilde{X}}_{k}^{i}$ denotes the position of landmark *i* in the global coordinate system.

Then, the states of both static and dynamic objects are calculated by newly detected landmark positions as denoted in Equation (17).

## 3. Evaluation

### 3.1. Environment Settings

The evaluation is conducted to compare the proposed algorithm to classical 1-point RANSAC algorithm. KITTI odometry dataset [39] is used to evaluate the performance of the classic 1-point RANSAC assuming static environments and modified 1-point RANSAC assuming dynamic environment. The test is conducted under the assumption that the state transition model follows a bicycle model, considering two configurations: (1) incorporating the control inputs into the augmented state vector for joint estimation, and (2) treating them as measurements. In the first configuration, the control inputs are included in the augmented state vector, which is denoted as $\xi_{k}≜{\left[x,y,\psi ,V,\delta \right]}_{k}^{⊤}$. In the second configuration, control inputs are treated as measurements. The control input vector $u_{k}≜{\left[V,\delta \right]}_{k}^{⊤}$ consists the longitudinal velocity *V* and the steering angle δ of the vehicle. Gaussian noise is also added to the velocity and steering angle to model sensor uncertainty for the test. The test is conducted for 200 frames of 11 sequences. Among the 11 sequences, sequences 1, 4, 8 consist dynamic objects and other sequences do not consist dynamic objects.

To evaluate the local accuracy of the proposed VO method, we adopt the Relative Pose Error (RPE) as an evaluation metric. The RPE quantifies the discrepancy between the estimated and ground-truth relative motions by comparing frame-to-frame transformations.

All parameters, including the EKF process and measurement noise covariances, RANSAC thresholds, and segmentation settings, were fixed across all experiments to ensure fair comparison. Only the initial state of the ego-pose was changed depending on the starting frame of each sequence.

### 3.2. Evaluation Using an Augmented State Vector Including Control Inputs

**Figure 4:**
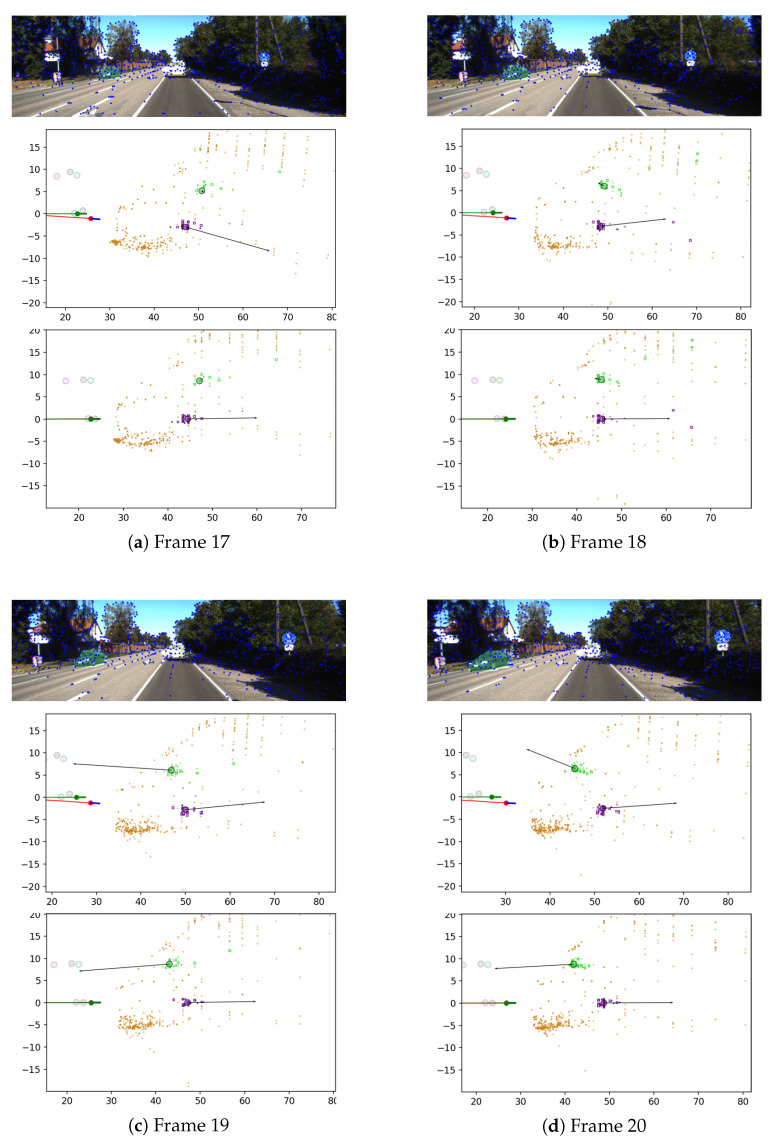
Estimation results for Frames 17–20 of Sequence 4. Top: input image; Middle: estimation with control inputs as states; Bottom: with control inputs as measurements. Ego-pose is shown as a circle with a short line indicating heading; static landmarks are brown dots; dynamic object landmarks are colored squares (green = approaching, purple = receding); and arrows indicate estimated object velocities.

Evaluation is first conducted using an augmented state vector including control inputs. Sequence 4, which was collected on a wide highway with dynamic vehicles, is presented as a representative example that demonstrates the effectiveness of the proposed method in dynamic environments. Figure 4 represents the comparison of the estimation results when control inputs are estimated as states (middle) and when they are used as measurements (bottom). The ego-pose is represented by a circle without an outline, indicating the agent’s position, and a short line, indicating its orientation. The agent’s trajectory is depicted as a line following its motion. Landmarks are plotted as small dots: inliers are shown as opaque points, while outliers are shown as translucent ones. In addition, the raw landmarks of dynamic objects are represented as squares. The distinction between objects is expressed through color differences in the landmarks. The positions of objects are indicated by circles, and the velocities of dynamic objects are visualized using arrows.

The scene includes one vehicle approaching the agent (green), another moving away from it (purple), and static landmarks distributed throughout the environment (brown). Since only a few static landmarks are available and they are difficult to match, assuming a static environment and applying the classical method can result in significant estimation errors or even lead to divergence. Although the absence of known control inputs during estimation makes the estimation highly sensitive to noise, the proposed method produces a coarse but informative estimate of object states that helps reduce ego-pose errors during estimation.

**Figure 5:**
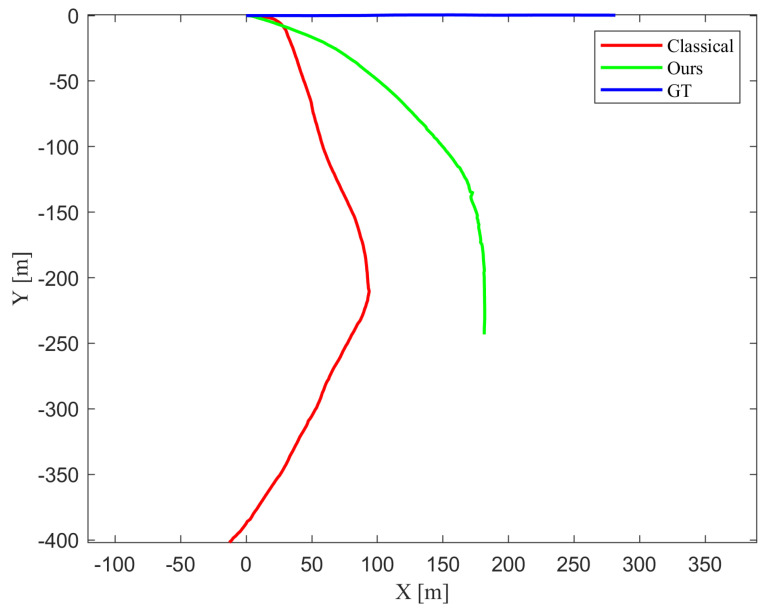
Comparison of estimated trajectories using the classical 1-point RANSAC (red), the proposed method (green), and the ground truth (blue). The proposed method jointly estimates control inputs as part of the state vector and produces a trajectory that more closely follows the ground truth, demonstrating improved accuracy and robustness over the classical approach.

Figure 5 shows the estimated trajectory. Although the proposed method is not free from estimation errors, it demonstrates significantly better robustness compared to the classical approach, which suffers from severe scale drift and larger error in orientation estimation. This result confirms that incorporating the motion of dynamic objects into the estimation process helps compensate for ego-pose errors.

**Figure 6:**
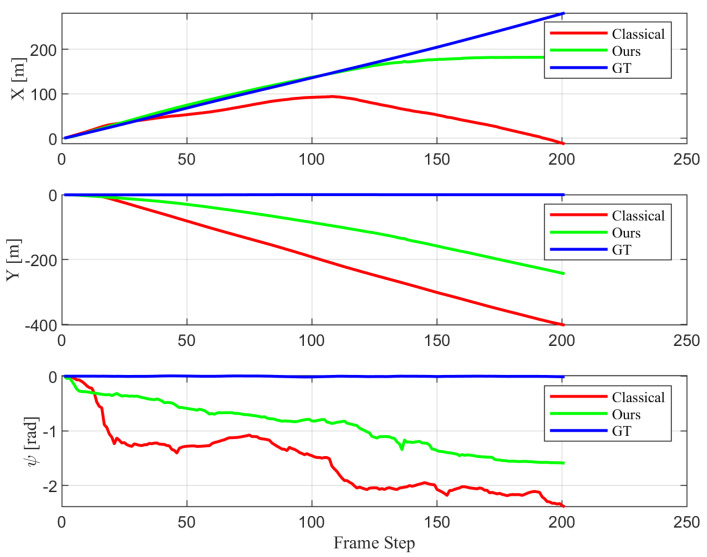
Comparison of ego-pose estimation using the classical 1-point RANSAC (red), the proposed method with control inputs in the state vector (green), and the ground truth (blue). Top, middle, and bottom plots show *s*, *y*, and yaw angle ψ over time, respectively. The proposed method achieves higher accuracy and stability in dynamic scenes by jointly estimating control inputs as part of the state.

Figure 6 shows the estimated ego-pose state values, where the proposed method exhibits less perturbation in estimation of orientation and less scale drift.

**Figure 7:**
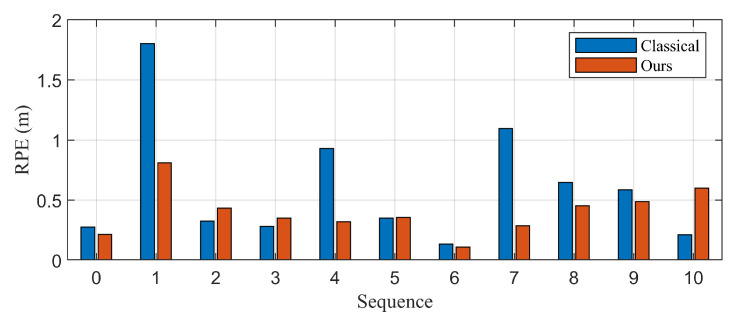
Quantitative evaluation of the proposed method, which incorporates control inputs into the state vector, using RPE on KITTI sequences. Significant improvements are observed in dynamic environments (Sequences 1, 4, and 8), with up to 53% lower error compared to the classical method. While performance in static environments remains comparable overall, noise-induced misclassification of static objects can slightly degrade accuracy.

Table 1 and Figure 7 further confirm the effectiveness of the proposed method. A substantial reduction in RPE is observed in dynamic sequences, achieving up to 53% improvement over the classical method. While performance in static environments is mostly comparable, the proposed method demonstrates strong robustness in handling dynamic objects through joint estimation of control inputs and states.

### 3.3. Evaluation Using Control Inputs as Measurements

**Figure 8:**
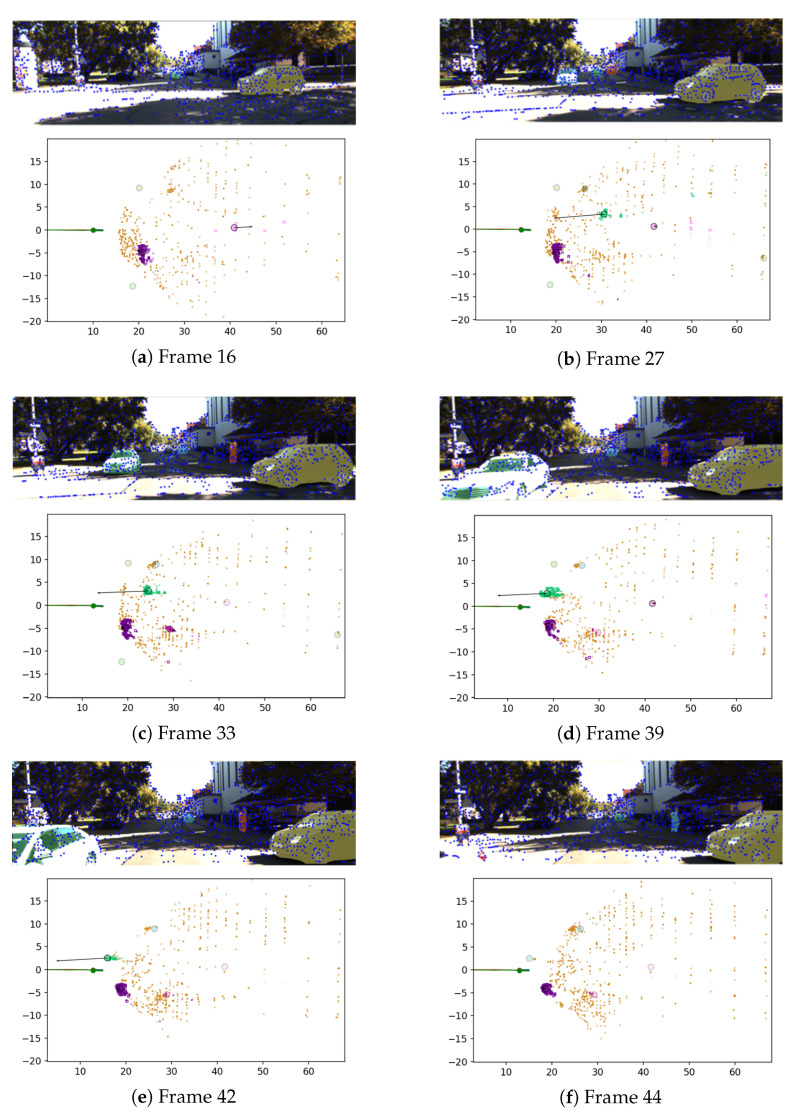
Estimation results for six selected frames (Sequence 8). Top: input images with detected features and instance segmentation masks. Bottom: estimated ego-pose and object states obtained by the proposed method. The ego-pose is shown as a circle with a short line indicating heading. Static landmarks are plotted as brown dots, and dynamic object landmarks are colored squares (green = approaching object, pink = receding object). Arrows represent the estimated object velocities, and circles indicate each object’s current position. The figure demonstrates that the proposed algorithm successfully detects and tracks dynamic objects across multiple frames while maintaining consistent ego-pose estimation.

The evaluation was also conducted by treating the control inputs as measurements. Figure 8 presents the estimation results of six selected frames from sequence 8.

For each frame, the upper figure shows the result of image-based measurement, including the detected features and instance segmentation masks. The lower figure shows the result of estimation.

Beginning with Frame 16 (Figure 8a), a dynamic object (pink) is present and is moving away from the agent. It is shown that the state of this moving object is successfully estimated.

In Frame 27 (Figure 8b), the dynamic object detected in Frame 16 remains dynamic in reality but is considered an outlier due to a large innovation, caused by having few landmarks and being too far from the agent. Instead, another dynamic object (green) appears, which is approaching the agent, and its state is also correctly estimated.

This dynamic object is consistently tracked in the subsequent frames. In Frames 39 (Figure 8d) and 42 (Figure 8e), the object state is shown to be estimated with minimal center shift caused by truncation during the filtering process.

Finally, in Frame 44 (Figure 8f), when the object is no longer visible in the image, its landmarks are excluded from the ego-pose estimation.

**Figure 9:**
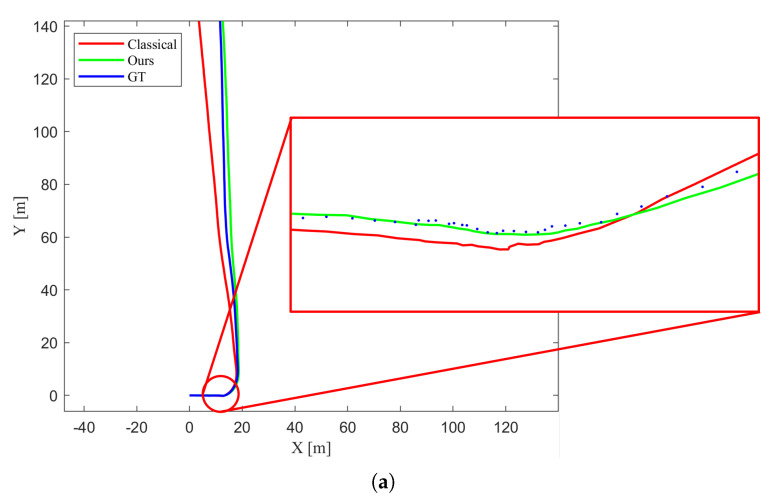

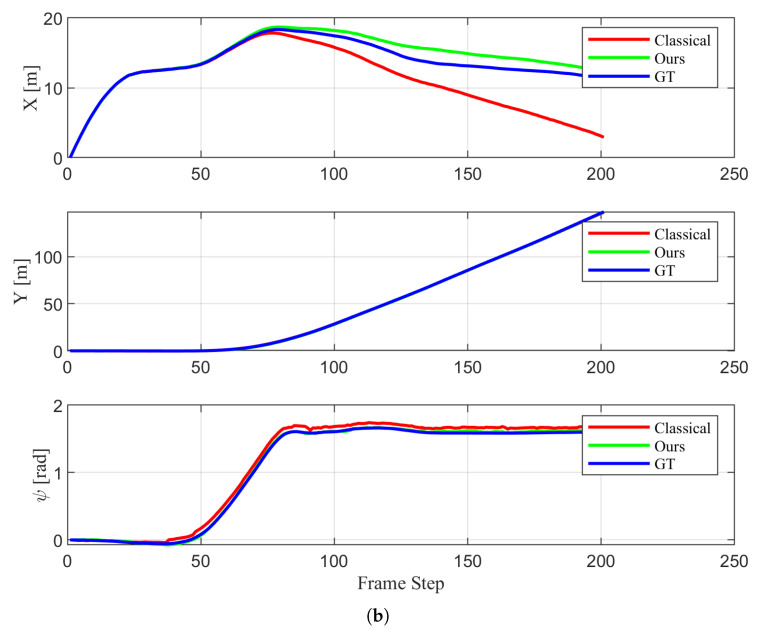
(**a**) Comparison of estimated trajectories using the classical 1-point RANSAC (red), the proposed method treating control inputs as measurements (green), and the ground truth (blue). The proposed measurement-based method produces a trajectory that more closely follows the ground truth, demonstrating improved accuracy and stability compared to the classical approach. (**b**) Comparison of ego-pose estimation over time using the classical 1-point RANSAC (red), the proposed method treating control inputs as measurements (green), and the ground truth (blue). Top, middle, and bottom plots show *X*, *Y*, and yaw angle ψ over time, respectively. Larger estimation errors are observed in the classical method when dynamic objects are present, while the proposed algorithm maintains better accuracy and smoother orientation estimation.

**Figure 10:**
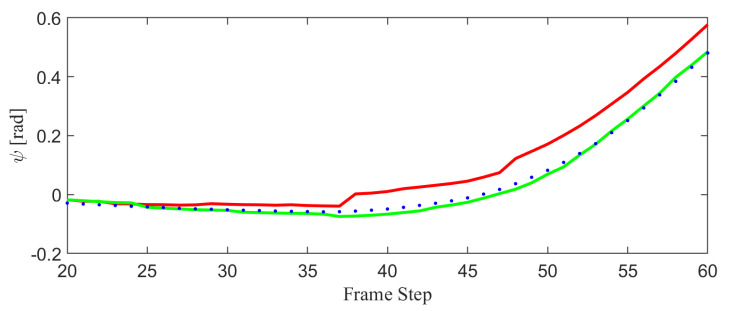
Yaw angle estimation at dynamic frames for the classical 1-point RANSAC (red), the proposed method treating control inputs as measurements (green), and the ground truth (blue). The red curve shows larger fluctuations in yaw estimation under dynamic conditions, while the proposed method (green) maintains a smoother and more stable estimate that closely follows the ground truth. This demonstrates the improved robustness of the proposed approach against disturbances caused by moving objects in the scene.

Figure 9 represents the estimated trajectories by classical 1-point RANSAC and the proposed method and Figure 9 represents the ego-pose values throughout time. Figure 9 show that the trajectory estimated by the proposed method follows the ground truth more closely than that of classical 1-point RANSAC. In Figure 9, bigger error is observed when using classical 1-point RANSAC than proposed algorithm for frames that exist dynamic objects. Figure 10 shows the yaw angle of the agent at dynamic frames in detail. It is observed that the yaw angle is estimated with less perturbation when used the proposed method.

**Figure 11:**
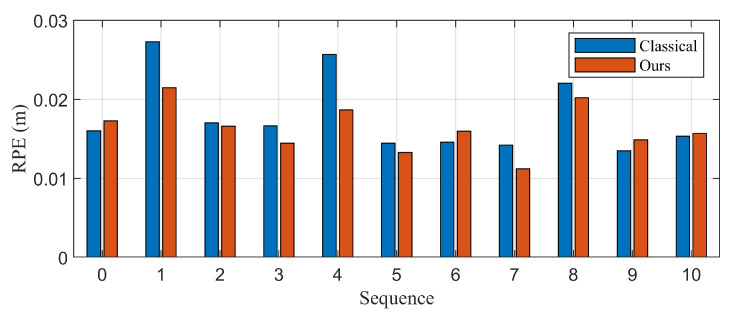
Quantitative evaluation of the proposed method using relative pose error (RPE) on KITTI sequences. Significant improvements are observed in dynamic environments (Sequences 1, 4, and 8), with up to 20% lower error compared to the classical method. While performance in static environments remains comparable overall, noise-induced misclassification of static objects can slightly degrade accuracy.

The evaluation result of the proposed algorithm is presented in Table 2 and Figure 11. In sequences 1, 4, 8, that contain dynamic objects, lower relative error is obtained with the proposed algorithm. There is slight variations in error in static environments, but the overall performance is nearly identical. 9% lower error is obtained for total sequences, and 20% lower error is obtained for sequences of dynamic environments. There are some factors that improve performance and others that degrade it at static environments. Additional filtering procedure that reject high-innovation landmarks can contribute to the performance of the estimation. In contrast, static objects can be misclassified as dynamic objects by the noise of the measurement, and it can cause decrease in performance of the estimation.  

## 4. Conclusions

We proposed a novel EKF-based visual odometry method designed for dynamic environments, where dynamic landmarks are explicitly incorporated into the ego-pose estimation process. Unlike conventional approaches that discard or ignore dynamic elements, our method leverages a 1-point RANSAC-based EKF framework, enabling robust state estimation even in the presence of moving objects and noisy measurements.

The proposed method demonstrated consistent improvements in relative pose error across various dynamic scenarios, validating its effectiveness in maintaining accurate ego-pose estimation under real-world conditions. We showed that the algorithm can estimate and track both ego-motion and object states without significant degradation caused by occlusion, landmark sparsity, or truncation artifacts.

Moreover, the framework is computationally efficient, achieving an average computation time of 0.0303 s per frame for the Kalman filter and 0.158 s per frame for image processing (feature matching and instance segmentation). This makes the system suitable for real-time applications and practical deployment in autonomous navigation tasks. The proposed method can be extended to various mobile platforms such as autonomous vehicles, drones, or robots operating in dynamic environments, where the assumption of static surroundings frequently fails.

While the method demonstrates reliable performance in typical dynamic scenarios, it may face challenges in extremely highly dynamic scenes or when measurements are severely degraded by noise. As part of future work, we plan to enhance the filter’s robustness in such conditions by incorporating motion priors and adaptive noise models that can better handle large landmark uncertainty and fast object motion. These improvements are expected to further increase the accuracy and reliability of ego-pose estimation in complex real-world environments.

## Figures and Tables

**Table 1:** Relative Pose Error on KITTI sequences with control inputs estimated as states.

Sequence	Classical	Proposed
00	0.2754	0.2130
**01**	**1.8018**	**0.8112**
02	0.3248	0.4334
03	0.2791	0.3508
**04**	**0.9288**	**0.3193**
05	0.3511	0.3559
06	0.1342	0.1094
07	1.0964	0.2852
**08**	**0.6455**	**0.4526**
09	0.5847	0.4866
10	0.2120	0.5997
Mean	0.6031	0.4016
Dynamic cases	1.1254	0.5277

**Table 2:** Relative Pose Error on KITTI sequences with control inputs as measurements.

Sequence	Classical	Proposed
00	0.0160	0.0173
**01**	**0.0273**	**0.0214**
02	0.0170	0.0166
03	0.0167	0.0144
**04**	**0.0257**	**0.0187**
05	0.0145	0.0133
06	0.0146	0.0160
07	0.0142	0.0112
**08**	**0.0220**	**0.0202**
09	0.0135	0.0149
10	0.0153	0.0157
Mean	0.0179	0.0163
Dynamic cases	0.0250	0.0201

## Data Availability

The data used in this study are openly available at the KITTI Vision Benchmark Suite (http://www.cvlibs.net/datasets/kitti/ (accessed on 17 October 2025)).

## Supplementary Materials

Video S1: EKF-based visual odometry in dynamic environments using modified 1-point RANSAC. The proposed method is available at: https://youtu.be/YT0lMQSoG2M (accessed on 17 October 2025).

## Appendix A. Classical 1-Point RANSAC

Algorithm of 1-Point RANSAC is described in Algorithm A1.

**Algorithm A1** 1-Point RANSAC-Based EKF Pose Estimation
**Input:** Predicted state and covariance ${\hat{\chi }}_{k|k-1}$, $P_{k|k-1}$; valid feature set $J_{k}$, innovation threshold $\gamma_{\mathrm{innov}}$, number of RANSAC hypotheses $n_{\mathrm{hyp}}$ **Output:** Updated state ${\hat{\chi }}_{k|k}$, covariance $P_{k|k}$, best inlier set $I_{k}$ Initialize best inlier set $\tilde{I}\leftarrow \emptyset$ **For** *h* = 1 to $n_{\mathrm{hyp}}$ **do** Randomly select one landmark $z_{k}^{i}$ from $J_{k}$ Perform temporary EKF state update using $z_{k}^{i}$ (no covariance update) $\Rightarrow {\hat{\chi }}_{k}^{h}$ Initialize temporary inlier set $I^{\left(h\right)}\leftarrow \left\{i\right\}$ **for** each $j\in J_{k}$, j≠i **do** Predict measurement ${\hat{z}}_{k}^{j}$ from state ${\hat{\chi }}_{k}^{\left(h\right)}$ Compute innovation $\nu_{j}=z_{k}^{j}-{\hat{z}}_{k}^{j}$ **if** $∥\nu_{j}∥<\gamma_{\mathrm{innov}}$ **then** Add *j* to inlier set $I^{h}$ **end if** **end for** **if** $\left|I^{h}|>|\tilde{I}\right|$** then** Update $\tilde{I}\leftarrow I^{h}$ and save ${\hat{\chi }}_{k}^{h}$ **end if** **end for** Perform full EKF update using inlier set $\tilde{I}$ Stack all residuals ν and Jacobians H for inliers Compute $S=HP_{k|k-1}H^{⊤}+R$ Compute Kalman gain $K=P_{k|k-1}H^{⊤}S^{-1}$ Update state: ${\hat{\chi }}_{k|k}={\hat{\chi }}_{k|k-1}+K\nu$ Update covariance: $P_{k|k}=\left(I-KH\right)P_{k|k-1}$ **for** each $i\notin \tilde{I}$ **do** Predict ${\hat{z}}_{k}^{i}$ from updated state ${\hat{\chi }}_{k|k}$ Compute innovation $\nu_{i}$, innovation covariance $S_{i}$ Compute Mahalanobis distance $d_{i}=\nu_{i}^{⊤}S_{i}^{-1}\nu_{i}$ **if** $d_{i}<\gamma_{\chi^{2}}$ **then** Add *j* to final inlier set $I_{k}$ **end if** **end for** **return** Updated state ${\hat{\chi }}_{k|k}$, covariance $P_{k|k}$, inlier set $I_{k}$

## Appendix B. RANSAC-Based Euclidean Transform Estimation

Algorithm of RANSAC-based Euclidean Transform Estimation is described in Algorithm A3.

**Algorithm A2** compute_rigid_transform: Least-Squares Rigid Transformation Estimation
**Input:** Two corresponding 2D point sets ${\overset{´}{X}}_{k}$ and ${\overset{´}{X}}_{k-1}$ (each with $N_{p}$ points) **Output:** Rotation matrix Ω∈SO(2), translation vector $\tau \in R^{2}$ Compute the centroid (mean) of each point set: $$m_{k}=\frac{1}{N_{p}}\sum_{i=1}^{N_{p}}{\overset{´}{X}}_{k}^{i},\;m_{k-1}=\frac{1}{N_{p}}\sum_{i=1}^{N_{p}}{\overset{´}{X}}_{k-1}^{m(i)}$$ Center the points by subtracting the centroids: $${\tilde{a}}_{i}={\overset{´}{X}}_{k}^{i}-m_{k},\;{\tilde{b}}_{i}={\overset{´}{X}}_{k-1}^{m(i)}-m_{k-1}$$ Compute the covariance matrix: $$\Theta =\sum_{i=1}^{N_{p}}{\tilde{a}}_{i}{\tilde{b}}_{i}^{⊤}$$ Perform singular value decomposition (SVD) on Θ: $$\Theta =U\Sigma V^{⊤}$$ Compute the rotation matrix: $$\Omega =VU^{⊤}$$ **if**det(Ω)<0 **then** Negate the second row of V and recompute Ω **end if** Compute the translation vector: $$\tau =m_{k-1}-Rm_{k}$$ **return** Ω and τ

**Algorithm A3** estimate_rigid_transform_ransac: Deterministic RANSAC-based 2D Rigid Transformation Estimation
**Input:** Two 2D point sets ${\overset{´}{X}}_{k}$, ${\overset{´}{X}}_{k-1}$ with $N_{p}$ points, reprojection threshold *T*, maximum iterations $N_{\max}$ **Output:** Best transformation matrix M∈SE(2), inlier mask $I\in {\left\{0,1\right\}}^{N_{p}}$ Initialize best inlier count to zero **for** each unique 2-point combination from ${\overset{´}{X}}_{k}$ and ${\overset{´}{X}}_{k-1}$ (up to $N_{\max}$ iterations) **do** Select two point pairs as a minimal sample set Estimate transformation (Ω,τ) using Algorithm A1 (compute_rigid_transform) Apply (Ω,τ) to all points in ${\overset{´}{X}}_{k}$ Compute reprojection error for each transformed point Determine inliers where error <T **if** current inlier count > best inlier count **for** Update best transformation M and inlier mask I **end if** **end for** **return** Best transformation M and inlier set I

## Appendix C. Calculation of Object State

A method of calculation of object state is represented in Algorithm A4.

**Algorithm A4** Adaptive RANSAC-based Velocity and Rotation Estimation
**Input:** Current points ${\overset{´}{X}}_{k}$, previous points ${\overset{´}{X}}_{k-1}$ **if** $|{\overset{´}{X}}_{k}|\geq 3$ **then** Estimate rigid transform M using Algorithm A3 (estimate_rigid_transform_ransac) between ${\overset{´}{X}}_{k-1}$ and ${\overset{´}{X}}_{k}$ Compute reprojection error for each transformed point cloud Construct inlier mask from RANSAC result **if** inliers exist **then** Apply DBSCAN to inlier points Extract largest cluster and update inlier mask **end if** **if** M≠∅ and valid inliers exist **then** Compute covariance matrix of inlier points **if** maximum eigenvalue of covariance $<T_{cov}$ **then** Compute cluster center $c=\mathrm{mean}({\overset{´}{X}}_{k}^{\mathrm{inlier}})$ Compute velocity from M $\dot{c}=\frac{\tau }{\Delta t}$ Extract rotation matrix Ω from M Compute angular velocity $\dot{\psi }=\frac{\arctan2(R_{21},R_{11})}{\Delta t}$ **else** Reject the object as outlier **end if** **else** Reject the object as outlier **end if** **else** Reject the object as outlier **end if** **return** inlier mask, center c, velocity $\dot{c}$, angular velocity $\dot{\psi }$

## Appendix D. Measurement Noise Covariance in Polar Coordinates

This appendix derives the measurement noise covariance in polar coordinates (ρ,θ) under the assumption that the stereo matching pixel disparity Δv and pixel location *v* have independent, constant variance $\sigma_{v}^{2}$.

Given a stereo camera with baseline *B* and focal length Φ, the depth *X* is computed from the disparity Δv as:

(A1) $$X=\frac{\Phi B}{\Delta v}$$

The horizontal pixel location *v* determines the offset from the optical center. The corresponding 3D point in Cartesian coordinates is given by:

(A2) $$\begin{array}{cc} x & =X=\frac{\Phi B}{\Delta v} \end{array}$$

(A3) $$\begin{array}{cc} y & =X\cdot \frac{v}{\Phi }=\frac{Bv}{\Delta v} \end{array}$$

The polar coordinates (ρ,θ) are defined by:

(A4) $$\begin{array}{cc} \rho & =\sqrt{x^{2}+y^{2}}=\frac{B}{\Delta v}\sqrt{\Phi^{2}+v^{2}} \end{array}$$

(A5) $$\begin{array}{cc} \theta & =\arctan\left(\frac{y}{x}\right)=\arctan\left(\frac{v}{\Phi }\right) \end{array}$$

Assuming that the measurement vector $z={\left[v,\Delta v\right]}^{T}$ has isotropic noise, we define the covariance as:

$$\mathrm{Cov}\left(z\right)=\sigma_{v}^{2}\cdot I_{2\times 2}$$

The measurement noise in (ρ,θ) is obtained via first-order error propagation using the Jacobian $J=\frac{\partial (\rho ,\theta )}{\partial (v,\Delta v)}$. Computing the partial derivatives:

(A6) $$\begin{array}{cc} \frac{\partial \rho }{\partial v} & =\frac{Bv}{\Delta v\sqrt{\Phi^{2}+v^{2}}},\;\frac{\partial \rho }{\partial \Delta v}=-\frac{B\sqrt{\Phi^{2}+v^{2}}}{{\left(\Delta v\right)}^{2}} \end{array}$$

(A7) $$\begin{array}{cc} \frac{\partial \theta }{\partial v} & =\frac{\Phi }{\Phi^{2}+v^{2}},\;\frac{\partial \theta }{\partial \Delta v}=0 \end{array}$$

Then, the variances in polar coordinates are:

(A8) $$\begin{array}{cc} \mathrm{Var}(\rho ) & =\sigma_{v}^{2}\left({\left(\frac{\partial \rho }{\partial v}\right)}^{2}+{\left(\frac{\partial \rho }{\partial \Delta v}\right)}^{2}\right) \end{array}$$

(A9) $$\begin{array}{cc} \; & =\sigma_{v}^{2}\left(\frac{B^{2}v^{2}}{\Delta v^{2}\left(\Phi^{2}+v^{2}\right)}+\frac{B^{2}\left(\Phi^{2}+v^{2}\right)}{{\left(\Delta v\right)}^{4}}\right) \end{array}$$

(A10) $$\mathrm{Var}\left(\theta \right)=\sigma_{v}^{2}\cdot {\left(\frac{\Phi }{\Phi^{2}+v^{2}}\right)}^{2}$$

To interpret these results, we express the variances in terms of (ρ,θ).

From the definition of ρ:

$$\rho =\frac{B}{\Delta v}\sqrt{\Phi^{2}+v^{2}}\;\Rightarrow \;\rho^{2}=\frac{B^{2}\left(\Phi^{2}+v^{2}\right)}{{\left(\Delta v\right)}^{2}}$$

Using this, we can express the second term in Var(ρ) as:

$$\frac{B^{2}\left(\Phi^{2}+v^{2}\right)}{{\left(\Delta v\right)}^{4}}=\frac{\rho^{4}}{B^{2}}$$

Thus, Var(ρ) becomes:

(A11) $$\mathrm{Var}\left(\rho \right)=\sigma_{v}^{2}\left(\frac{v^{2}}{\Phi^{2}+v^{2}}\cdot \frac{B^{2}}{\Delta v^{2}}+\frac{\rho^{4}}{B^{2}}\right)$$

This shows that Var(ρ) includes a term proportional to $\rho^{4}$, and as depth increases (i.e., Δv→0), the second term dominates. Therefore, in the high-depth regime, radial uncertainty approximately grows with the fourth power of range:

(A12) $$\mathrm{Var}\left(\rho \right)\approx \sigma_{v}^{2}\cdot \frac{\rho^{4}}{B^{2}}$$

For the angular variance, we express *v* in terms of θ:

$$\frac{v}{\Phi }=\tan\theta \;\Rightarrow \;\Phi^{2}+v^{2}=\Phi^{2}\left(1+\tan^{2}\theta \right)=\frac{\Phi^{2}}{\cos^{2}\theta }$$

Substituting into the expression for Var(θ) yields:

(A13) $$\mathrm{Var}\left(\theta \right)=\sigma_{v}^{2}\cdot {\left(\frac{\Phi }{\Phi^{2}+v^{2}}\right)}^{2}=\sigma_{v}^{2}\cdot \frac{\cos^{4}\theta }{\Phi^{2}}$$

This indicates that angular uncertainty is minimized near the image center (θ≈0) and increases toward the periphery, as $\cos^{4}\theta$ decreases.
